# Way out of the one-way? Effects of the COVID-19 pandemic on the generation of waste from packaging in Germany

**DOI:** 10.1007/s00550-022-00525-z

**Published:** 2022-03-02

**Authors:** Elisabeth Süßbauer, Henning Wilts, Sarah Julie Otto, Jennifer Schinkel, Klara Wenzel, Rabea-Lorina Dehning, Justus Caspers

**Affiliations:** 1 Center for Technology and Society, Technische Universität Berlin, Berlin, Germany; 2 Division Circular Economy, Wuppertal Institute for Climate, Environment, Energy, Wuppertal, Germany; 3 Department of Circular Economy and Recycling Technology, Technische Universität Berlin, Berlin, Germany; 4 Department of Sustainable Engineering, Technische Universität Berlin, Berlin, Germany

## Introduction

Next to some positive environmental impacts of the COVID-19 pandemic, like reduced mobility, it also brought negative ones. One of the negative outcomes is an alarming increase in waste plastics and a substantial decrease in its recycling on a global basis (Memon 2021; Kumar et al. 2021; Brock 2020). Main sources of increased waste plastics are related to the use of plastics in medical and food packaging (Leal Filho et al. 2021).

Even before the pandemic, Germany has been considered as one of the main packaging waste producers within the European Union: more than 18 million tons are generated annually (Schüler 2020). This corresponds to 227.5 kg per inhabitant while, for example, Croatia produces only 67.8 kg per inhabitant per year (eurostat 2020). The food sector contributes especially to the packaging waste of private consumers: around 60% of the consumers’ packaging waste stems from packaging for beverages, food and pet food (Schüler 2020). Measures introduced to contain the COVID-19 pandemic since March 2020, in particular the closure of shops, restaurants, and cafés as well as day-care centres and schools, have had different effects on the volume and distribution of packaging waste in Germany.

Four developments have particularly influenced the use of single-use packaging in the food sector since the first lockdown^1^ in March and April 2020. First, consumer behaviour regarding nutrition and leisure activities have changed; for example, cooking and eating at home became more common (Deloitte 2021; Busch et al. 2020; Kumar and Rasquin 2020). Second, many companies stopped accepting reusable containers for coffee and food (DUH 2020) while, at the same time, consumers were more concerned about hygiene of unpackaged food products (Süßbauer et al. 2020a; Böhm et al. 2021). An indicator of being concerned about unpackaged food is that, according to a representative survey among German consumers, 44% of them touched less products when shopping in a grocery store (Statista 2020a). Third, food and prepared dishes are increasingly being bought online (Deloitte 2021; Statista 2020b; BMU 2020; bitkom 2020; Busch et al. 2020). However, compared to the overall increase of online shopping by 48% during the pandemic, the share of online ordering of fruit and vegetables is still relatively small (0.9%) (Fruchthandel Online 2021). Fourth, people bought or ordered more prepared food for take-away (Deloitte 2021).

These changes in consumer behaviour continued until now and may continue despite the relaxation or stop of COVID-19 countermeasures, because habits might turn into household practices that are, for example, integrated in the temporal organisation of everyday life (Greene et al. Forthcoming o.J.). Such long-term changes in attitudes and behaviours can, firstly, impact points and routes of disposal and, secondly, influence the total amount of packaging waste that is generated. Furthermore, these changes have consequences for actors whose business model or organisational goal is to avoid disposable packaging, e.g., suppliers of systems for reusable packaging, zero-waste shops or consumer advice. Considering these developments, the following questions arise:

- How do recent changes in consumption in the wake of the COVID-19 pandemic affect the avoidance of packaging waste?
- How can an increase in packaging waste be countered and the previous trend towards unpackaged and reusable solutions be revived and promoted?

**Fig. 1:**
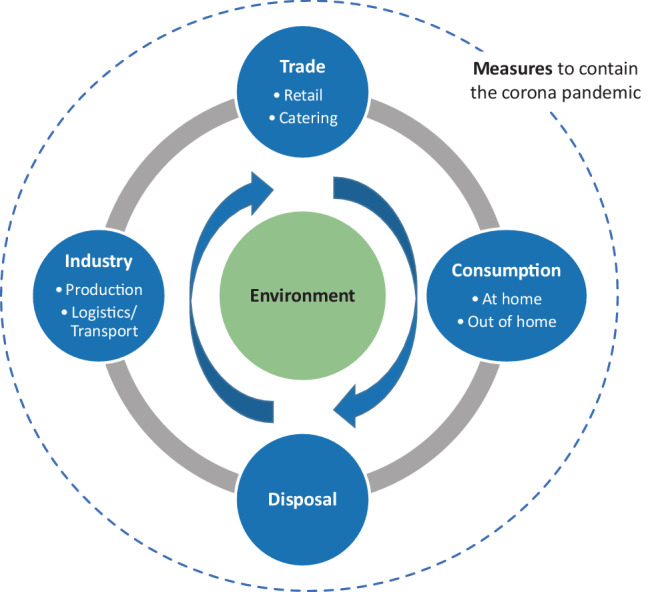
Central stakeholder groups of the food packaging system

To tackle these questions, we use a systemic approach that regards packaging as a network of interrelated interests of industry (manufacturing and logistics), trade (retail and catering), consumers and the waste management sector (see Fig. 1). To analyse this network, we applied three methods. First, we analysed secondary sources such as surveys. Second, we conducted semi-structured interviews with seven actors from industry, consumer education and waste management in May and June 2020. Third, we used the questions from the interview guideline to do an online survey among representatives of the public waste management industry. The survey was carried out in cooperation with *Akademie Dr. Obladen*, a trainer for organisations of the municipal waste management, in the period of 29 June to 3 July 2020. However, since the period of data collection was in July 2020, we did not have much time to prepare for this unforeseen situation. Thus, our empirical data is rather explorative. Moreover, there are still not many scientific sources or statistics available about the production of waste during the pandemic.

Based on the secondary analysis, we first describe the current status quo: the interrelated functional impacts of COVID-19 within the packaging system (Section 2). This is followed by an evaluation of the status based on the interviews and online-survey: what organisational and practical challenges, but also what learning experiences are reported from the perspective of different stakeholders (Section 3)? Based on these insights, we present the future risks of the COVID-19 crisis for waste prevention and formulate recommendations for action made by the interviewees (Section 4). Finally, conclusions from a research perspective are drawn (Section 5).

## Effects of COVID-19 on the German packaging system—shifts and interdependencies

The measures taken to contain the COVID-19 pandemic have probably led to a shift and/or strengthening trends in the food packaging sector. In this section, possible interactions between the measures and these trends are outlined and discussed. Four stakeholder groups are central since they mutually influence each other regarding the generation of packaging waste: industry, trade, consumption, and disposal (see Fig. 1). Within consumption, we distinguish between out of home and domestic consumption since developments of packaging waste differ in these two domains. In the following, the impact of the COVID-19 pandemic on the interactions between these stakeholder groups are described taking Germany as an example.

On the* industrial side*, during the first phase of the pandemic, sector-specific production of goods and raw materials, for example in the food and automotive sectors, was discontinued, which in turn had a strong impact on trade in Germany (Recyclingportal 2020). The logistics industry has faced a major challenge. Closed shops have not only increased online trade, but also the demand for packaging solutions: packaging materials that enable fast and hygienic packaging were, particularly in demand. The use of plastic bags was especially at the peak of this demand (e-commerce magazin 2020). A significant increase in the volume of corrugated cardboard packaging was also noted, as well as filling and barrier materials and packaging supplies such as tapes and mailing bags. However, a return to classic, stationary trade is expected as soon as the effects of the pandemic subside (klingele 2020).

Concerning *trade*, changes in consumer behaviour influenced commercial enterprises in the retail and catering sectors, which were caused by exit and access restrictions and the closure of commercial enterprises. For example, due to the prescribed contact and access restrictions, many catering businesses were forced to expand their range of services to include delivery services and take-away options for food and beverages, which has led to increased use of food delivery services (Röttig 2020). Subsequently, a shift in waste and packaging waste flows was observed (DGAW 2020). Furthermore, a shift of packaging volumes between industrial sectors themselves is plausible. The closure of mass catering establishments (e.g., refectories, canteens) and strengthening of take-away food retailing have led to reduced usage of bulk packs for food products, which are replaced by the consumption of smaller convenience packaging in food retail outlets. These show a higher generation of packaging waste in relation to the packaged goods.

For the *out of home consumption sector*, before the pandemic, several reusable alternatives had become established to reduce the use of disposable packaging within the food sector. These include reusable cups for drinks and beverages, reusable bowls, and pizza trays for take-away-food as well as reusable bags and nets for baking goods, fruits and vegetables. At present, however, these alternatives are being used less frequently due to hygiene concerns and (partly self-imposed) hygiene rules (Zentralverband des Deutschen Bäckerhandwerks e.V. 2020). Instead, one-way single-use packaging, especially made out of plastic, is increasingly being used again (EEA 2020). This has led to a further increase of improper disposal of single-use packaging in public spaces, especially when people were allowed to meet again in summer 2020 (UBA 2020; Betscka 2020).

Concerning *domestic consumption*, the growing preparation and consumption of food at home has led to an increase in household waste (Freyler 2020), while the amount of commercial waste has decreased (DGAW 2020). According to a recent survey by the Federal Association of the German Waste Management, Water and Raw Materials Industry, companies were already able to detect strong regional fluctuations in waste volumes during the first lockdown in March 2020. In individual cases, an increase of 20% was recorded for glass and light packaging waste from private households. Waste volumes from industry, trade and commerce at least declined or stopped accordingly. On an annual basis, the companies report an average increase of 5.9% in the amount of glass wastes. Light packaging made of plastics, metals and composite materials increased by 5.7% (BDE 2020). It can also be assumed that the increased demand and hoarding of reusable bottles, cans and frozen products instead of fresh products will lead to changes in composition, returns and packaging waste (Freyler 2020).

Concerning *disposal*, the described interactions and shifting effects have resulted in a change in waste volume and composition. Ultimately, these are consequences that the waste management industry, situated at the end of the value chain, must face. At times, statements were circulated about the decrease in sorting qualities of the household waste produced (DGAW 2020). At the end of March 2020, the German Ministry of Environment announced that households infected with the COVID-19 virus or in quarantine should temporarily not separate their waste as a precautionary measure (BMU 2020). Furthermore, the decline in sorting quality can be explained by the overall increase in household waste and uncertainties about hygiene requirements. Some of the household waste bins available did not offer sufficient capacity and encouraged the use of other waste containers (DGAW 2020). Changes in composition and delays in disposal, e.g., due to hoarding, caused a decreasing availability of secondary raw materials for specific material flows. However, the production of certain goods is highly dependent on such secondary raw materials, which occasionally led to bottlenecks, e.g., in the case of paper for packaging (EUWID 2020).

Overall, this preliminary descriptive analysis of developments and shifts in the food packaging sector suggests that the pandemic had various effects on the generation of waste from packaging in the different stakeholder groups that are interdependent from each other.

## Navigating COVID-19—practical challenges and learnings from stakeholders of the packaging system

The outlined complex interactions and their partly contradictory effects on the prevention of waste from packaging were further explored in interviews with the practice partners of the research group PuR and other relevant stakeholders within the packaging system.

### Method

A total of seven semi-structured interviews were conducted with stakeholders from the food retail and catering, consumer education and waste management sector in May and June 2020 (see Table 1). The interviews lasted between 41 and 77 min and included thirteen questions. The interview questions were about the subjective experiences of the stakeholders in their respective sector and addressed the following aspects: reactions and implemented measures adopted by the stakeholders to address the changes and challenges; lessons learned; perceived current changes among consumers with regard to (food) packaging; expected future changes in packaging consumption in Germany resulting from the pandemic; vision for packaging prevention; recommendations for measures for sustainable packaging avoidance. We have analysed the interviews according to the main categories of the interview guideline following the method of summarizing qualitative content analysis (Mayring 2015).

**Table 1:** Overview interview partners

Sector	Interviewee	Project partner
Food retail	Owner of zero-waste shop	Yes
Food catering	Food delivery service	Yes
Consumer education	Non-profit association	Yes
	Consumer advice centre	No
Waste management sector	Association of municipal waste companies	No
	Technical expert service for recycling of packaging	No
	Collecting system for packaging	No

Additionally, an online survey among 24 persons from the public waste management industry was carried out to validate opinions expressed by the interviewees.

In the following, the practical challenges and learnings of the interviewed stakeholders working in the field of packaging are presented and illustrated with examples.

### Experiences from the food retail and catering sector

In the food retail and food catering sector, we interviewed founders of a zero-waste shop as well as a manager of a food delivery service using reusable containers. With their business models, these two actors are pursuing the specific goal of avoiding packaging waste.

Both report that they initially had to struggle with a drop in sales. In the case of the zero-waste shop, this was due, among other things, to hygiene aspects in the stationary trade associated with the pandemic. In view of the zero-waste shop, the main problem was not the integration of the requirements into the shop concept, since they would already meet high hygiene requirements even before the pandemic (e.g., use of disinfectants and gloves), but the concerns of the customers concerning the safety of unpackaged food. The zero-waste shop adapted to these altered conditions by allowing customers to continue bringing their own containers, but these could only be filled by staff. For risk groups and those who preferred it, a pre-packing and delivery service was set up and integrated into the zero-waste shop. This was very well received by customers. However, the new delivery concept had to be developed within a very short time, which also had to guarantee the obligation to provide information on ingredients for reusable containers (e.g., via newly developed labels). The introduction of new processes involved additional time and personnel expenditure. On the other hand, an increased need for communication with customers was identified—also to create renewed trust. It was also noted that there were no concrete recommendations for environmentally friendly implementation of the corona measures in companies.

There were also adjustments made to the delivery service using reusable containers. Due to the closure of many businesses, the demand for food delivery to the workplace collapsed. For this practice partner, this circumstance led to the company adapting to the changed conditions and has since been delivering to private individuals. As a result, the delivery service now serves a much larger delivery area. In addition, delivery is now contactless, with push messages informing customers of their order, and this new feature has been very well received. However, the disadvantage of the new, adapted system is that fewer collective orders are placed, which has increased the transport distances, i.e., fewer marked routes can be taken.

However, these adjustments were also considered to be a valuable experience. In the zero-waste shop, for example, the company had already wanted to deal with a delivery concept before and was now forced to deal with the subject matter more quickly—the concept was put in place in just one week. Also, at the delivery service the experience was gained, that the company’s own employees were able to carry out a complete changeover of the system in the shortest possible time and without preparation. The strength of the team has proven itself through the crisis: Employees can familiarise themselves with other areas of responsibility and can be deployed flexibly. The already practised agile management of the start-up company has paid off.

### Consumer education experiences

In the field of consumer education, we interviewed a non-profit association that is committed to promoting the consumption of tap water and a representative from the consumer advice centres (“Verbraucherzentralen”) which offers information and advice regarding consumer related topics, e.g., waste prevention.

For the non-profit association on tap water, it was mainly the contact restrictions that have had a severe impact on everyday business: Berlin drinking fountains were not put into operation, events in schools, in day-care centres or on the association’s own premises had to be cancelled. This made it more difficult for the project to have a personal exchange with consumers as a direct target group. As a comparatively small organisation, however, it was possible to adapt relatively quickly to the new conditions. During the first lockdown, many offers were moved to the digital space (e.g., online training seminar for multipliers) and project resources were restructured. In addition, new processes and coordination loops became necessary and internal communication was more complex. The lack of informal exchange among colleagues in the office was challenging. Since the association had previously also relied heavily on digital communication (e.g., its own YouTube channel), the technical changeover was easy, but the association is still faced with the question of how the successful sensitisation of consumers to environmental issues can work in the future.

The consumer centres reported similar experiences. Questions they are concerned with now are: How to reach less “digitally-affine” target groups, e.g., elderly people or those with lesser education? How to maintain a personal relationship with consumers? Although the new circumstances have made time management more difficult in many projects, an acceleration of the implementation of already planned measures was also noticed here. For example, the online offer was strengthened within a short time. For the future, it is planned to strengthen both the personal contact with consumers and the digital offer.

### Experiences from the disposal sector

The COVID-19 pandemic was also associated with enormous challenges for those involved in waste management, which underlined the “systemic relevance” of the disposal sector. Despite all the requirements for keeping distances and complying with hygiene regulations, waste still had to be disposed of. In the online survey amongst relevant stakeholders in the waste management sector manifold concrete effects were indicated: it was mentioned that recycling centres had to be closed and additional safety measures for waste sorters had to be implemented. In addition, an increase in the volume of residual waste was observed, which is partly due to a change in separation behaviour. More than 50% of the survey participants expected a rise in the use of single-use packaging due to an increased need for hygiene and changed consumption patterns. As in many other areas, communication with citizens was also made considerably more difficult by the pandemic: campaigns on waste separation which were launched during this period were perceived as hardly noticed due to the current focus of the media. It also seems that the topic of waste prevention has currently, at least temporarily, been out of focus in many areas. It has also become apparent that in the waste management industry, digital processes will become more important in the future, e.g., online communication with customers.

## Strengthening packaging prevention—recommendations for action by stakeholders

**Fig. 2:**
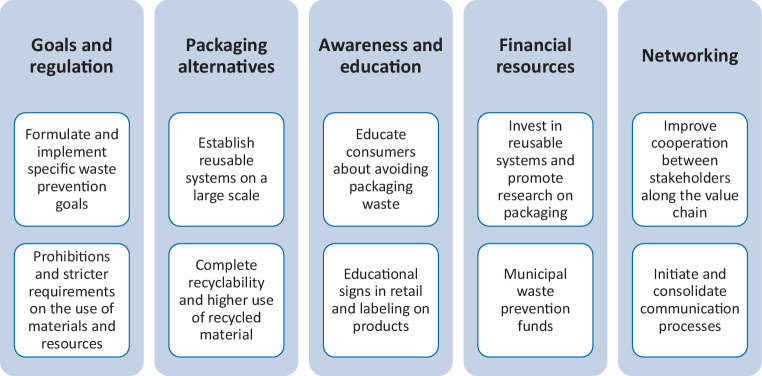
Recommendations for reducing the volume of packaging waste from the perspective of stakeholders

Next to the current practical experiences, we asked the interview partners what measures they think are needed to address the challenges posed by the COVID-19 pandemic and to effectively prevent packaging waste in the future. From the interviews with the actors from the retail and catering, consumer education and disposal sectors, we derived impulses and recommendations for actions that could contribute to strengthening food packaging waste prevention both in the short and long term. Their recommendations address various aspects (see Fig. 2), which will be presented in more detail below.

In most cases, the recommendations of the interviewees concern issues that already existed prior to the COVID-19 pandemic. A possible reason might be that the interviews were conducted at the beginning of the pandemic and the specific challenges that the pandemic might raise in the long term were not yet fully known. Another explanation could be that the pandemic has maintained or increased the urgency of already existing demands. It should also be noted that the plastic as well as the plastic packaging industry have to be considered as beneficiaries of the crises (see Brock 2020; Kumar and Rasquin 2020), especially because many concrete activities for plastic packaging waste prevention have been postponed or overruled as highlighted in Section 3. Some of the recommendations are demands on policy makers, such as the need for waste prevention goals, stronger regulation and increased financial support. Others refer to building knowledge, establishing networks, and strengthening cooperating along the value chain. Furthermore, interviewees requested establishing alternatives to the currently prevailing types of packaging.

### Anchoring prevention in concrete goals and regulations

A more ambitious waste prevention programme was identified by interviewees as necessary for the sustainable reduction of packaging waste, which should set clear targets for the prevention of packaging waste. This should make it possible to integrate packaging waste prevention into concrete planning, for example at local level, and to base strategic decisions on these targets. Furthermore, a regulatory intervention was also called for to reduce packaging waste. These ranged from stricter regulations for food packaging with a low relation of actual product to packaging volume, included requirements for the use of materials in packaging and a general ban on disposable packaging in the food sector. This ban should go beyond the EU Waste Framework Directive, which came into force in July 2021 and is limited to individual disposable products made of certain plastics (expanded polystyrene and oxo-degradable plastics).

### Promotion of resource-saving packaging alternatives

To reduce the generation of waste through disposable packaging, reusable solutions should be promoted both in online mail order and in stationary shopping and take-away meals, according to the interviewees. As the COVID-19 pandemic will likely serve as an accelerator for packaging trends already underway, such as the use of food delivery services, it should be ensured that reusable alternatives are actually used in a resource-saving way. As suggested by Andert (2020), this could include, e.g., regional delivery and collection as well as a long containers’ life. To ensure that these solutions make a relevant contribution to packaging waste prevention, interviewees suggested that reusable systems and unpackaged concepts should be established on a large scale. Furthermore, complete recyclability for food packaging and higher use of recycled materials in packaging were mentioned as important factors for packaging waste avoidance.

### Awareness raising and consumer education

When shopping and using take-away offers, consumers often face difficulties in identifying the most resource- and environmentally friendly alternative. During the COVID-19 pandemic, the challenge is compounded by the fact that reusable solutions are sometimes presented as hygienically questionable and rejected. When it comes to disposal, many consumers also face the problem of not knowing how to dispose of their packaging waste so that it can be recycled. In this respect, the interviewees pointed out that more information on the topics of packaging avoidance, materials used, hygiene aspects and waste separation is necessary to enable consumers to make informed decisions. The “raised forefinger”, however, was considered rather counterproductive and positive marketing should be preferred instead. In this context, appropriate information signs about unpackaged goods in supermarkets were also suggested as an effective measure. In an interview, one respondent also spoke out in favour of mandatory labelling for waste separation for more or all products.

### Provision of financial resources

The interviewees pointed out that financial resources are necessary to strengthen packaging waste prevention and achieve actual success. As a concrete starting point, improved financing of municipal waste management for prevention was proposed, e.g., through a joint fund. One possibility, according to the respondents, would be to invest in refill and returnable systems, as these are often not yet economical enough. Promoting research on sustainable packaging was also regarded as an important factor.

### Networking waste prevention actors

The interviewees also highlighted the need for networking among the relevant stakeholders. To this end, communication opportunities should be created and consolidated between the various actors along the value chain and civil society. As a possibility, it was suggested to establish a nationwide platform to also include municipal waste advisors and to network with them. Furthermore, stakeholders from the field of production would need to cooperate closely with stakeholders from the field of waste management.

## Conclusions and outlook—Packaging waste prevention in times of crisis

The preliminary description of the effects of COVID-19 on the volume and distribution of packaging waste shows that food packaging is a complex system that interacts with many factors and dimensions. Along the value chain, different stakeholders with different interests are involved in this system—beginning with the plastics and packaging industry who clearly benefited from the crisis due to low oil prices (Brock 2020). These strengthened path dependencies that just had started to become loosened will now again challenge future waste prevention activities.

We complemented the analysis with exploratory interviews and a small online survey with actors working in the field of packaging waste prevention (zero-waste shop, delivery system, consumer education and waste disposal) to contrast the descriptive analysis with the ongoing organisational practices that have developed during the pandemic. These qualitative flashlights indicate that the pandemic has put actors from the realm of practice to a tough test. At the same time, valuable learning experiences also result from the crisis. This simultaneity of risks and opportunities is associated with uncertainties about the progress of the pandemic and the resulting restrictions and changes in everyday life.

This complexity of the “packaging system” and the difficulty of estimating the further course of the pandemic make it very difficult to forecast changes in packaging waste volumes. However, the developments outlined above, and challenges experienced already in 2020 indicate that disposable plastic packaging could regain strength as it is designed to protect against infection. The problem will be particularly acute if consumers incorporate these changes into their everyday habits and, for example, prefer to buy fruit and vegetables in plastic packaging at the supermarket or order food that is extra packed to avoid contact with other people. However, in the German retail market, there have also been trends towards more demand and supply of unpackaged organic fruits and vegetables (e.g. Alnatura Produktions- und Handels GmbH 2020; Edeka Handelsgesellschaft Südwest mbH 2021). At this moment in time, it is still unclear how these developments will interact and if these trends can be attributed to different consumer groups. Thus, future studies should analyse impacts of the pandemic on packaging waste from a multidimensional perspective including stakeholders from the industry, trade, consumption, and disposal—our exploratory empirical data could be a starting point for this.

The perceived future risks and measures to react on the COVID-19 impacts outlined by the interviewed practitioners underlines that precycling—the consequent and systemic avoidance of single-use packaging—is still in its infancy and that prevention structures, such as reusable packaging systems and zero-waste shops, are still fragile. On the one hand, the COVID-19 crisis thus carries the risk that the efforts to reduce packaging waste that have been initiated in recent years will be slowed down or even reversed. On the other hand, such drastic cuts in the wake of the COVID-19 crisis could also be an opportunity to fundamentally rethink resource-intensive production and consumption patterns and to develop and establish sustainable systems.

The current situation, at least in Germany, could therefore be used not only to strengthen existing precycling concepts such as unpackaged products or reusable packaging, but also to establish sustainable and resilient waste avoidance structures in the packaging sector. Once build up, these structures should also function under changing conditions or crisis situations. It is certain that the pressure to act to avoid (plastic) waste, which was perceived by broad sections of the population before the COVID-19 crisis, will not automatically be restored—clear signals will be necessary here to be able to build on the successes achieved in the past. Another interesting aspect for future research will be what effects legal regulations such as the ban on certain single-use plastics or the intended promotion of reusable packaging in the take-away sector in the German Packaging Law have on the amount of packaging waste generated. Furthermore, a cross-country comparison could be an important next step to understand how COVID-19 has influenced precycling opportunities.

The junior research group PuR wants to contribute to a step in this direction. It is concerned with the question of how conducive framework conditions for packaging waste prevention should be established and adapted so that precycling solutions can become socially accepted. Because recycling alone cannot save us from the waste crisis; for a way out of the one-way, we still need good precycling ideas that preferably prevent packaging waste from arising in the first place.

## Footnotes

1 With the term “lockdown” we refer to measures to contain the spread of the COVID-19 virus that are related to the closure of restaurants, non-essential stores, kindergartens, schools as well as to contact and travel bans. These measures and the periods of the lockdowns differed slightly between countries (and federal states within countries), for example in some countries, like in Germany, people could leave the homes for doing sports or shopping, in some they were obliged to stay at home during certain periods of the day. In this article, we only refer to the German context.
